# CADM1 is a TWIST1-regulated suppressor of invasion and survival

**DOI:** 10.1038/s41419-019-1515-3

**Published:** 2019-03-25

**Authors:** Edward J. Hartsough, Michele B. Weiss, Shea A. Heilman, Timothy J. Purwin, Curtis H. Kugel, Sheera R. Rosenbaum, Dan A. Erkes, Manoela Tiago, Kim HooKim, Inna Chervoneva, Andrew E. Aplin

**Affiliations:** 1 Department of Cancer Biology, Thomas Jefferson University, Philadelphia, PA 19107 USA; 2 Sidney Kimmel Cancer Center at Jefferson, Philadelphia, PA 19107 USA; 3 Department of Pharmacology and Physiology at Drexel University College of Medicine, Philadelphia, PA 19102 USA; 4 Departments of Pathology, Anatomy, and Cell Biology, Thomas Jefferson University, Philadelphia, PA 19107 USA; 5 Division of Biostatistics in Pharmacology and Experimental Therapeutics, Thomas Jefferson University, Philadelphia, PA 19107 USA

## Abstract

Metastatic cancer remains a clinical challenge; however, patients diagnosed prior to metastatic dissemination have a good prognosis. The transcription factor, TWIST1 has been implicated in enhancing the migration and invasion steps within the metastatic cascade, but the range of TWIST1-regulated targets is poorly described. In this study, we performed expression profiling to identify the TWIST1-regulated transcriptome of melanoma cells. Gene ontology pathway analysis revealed that TWIST1 and epithelial to mesenchymal transition (EMT) were inversely correlated with levels of cell adhesion molecule 1 (CADM1). Chromatin immunoprecipitation (ChIP) studies and promoter assays demonstrated that TWIST1 physically interacts with the CADM1 promoter, suggesting TWIST1 directly represses CADM1 levels. Increased expression of CADM1 resulted in significant inhibition of motility and invasiveness of melanoma cells. In addition, elevated CADM1 elicited caspase-independent cell death in non-adherent conditions. Expression array analysis suggests that CADM1 directed non-adherent cell death is associated with loss of mitochondrial membrane potential and subsequent failure of oxidative phosphorylation pathways. Importantly, tissue microarray analysis and clinical data from TCGA indicate that CADM1 expression is inversely associated with melanoma progression and positively correlated with better overall survival in patients. Together, these data suggest that CADM1 exerts tumor suppressive functions in melanoma by reducing invasive potential and may be considered a biomarker for favorable prognosis.

## Introduction

Patients presenting with early stage cancers who undergo surgical intervention have a favorable overall survival. By contrast the prognosis for patients with metastatic disease is poor. Metastatic progression is a complex process that includes the ability to migrate and invade through the in situ organ, intravasate into vasculature, resist anoikis to survive in the bloodstream, and extravasate for colonization of a distant organ^1^. Associated with the initial migration and invasion, tumor cells need to alter a gene expression program, collectively referred to as epithelial-mesenchymal transition (EMT)^2^. TWIST1 is a transcription factor implicated in both developmental and pathological EMT^3,4^. TWIST1 contributes to an EMT-like phenotype switch in melanoma that enhances migratory and invasive function^5,6^. Our group has previously demonstrated that TWIST1 plays a role in the ability of melanoma cells to invade through the dermal layer in part by up-regulating the matrix metalloprotease, MMP-1^7^. However, the range of TWIST1 targets is poorly characterized.

Dysregulation of cell-cell junctions is an important aspect of pathological EMT^8^, and TWIST1 have been demonstrated to contribute to this process^2,8^. The cell adhesion molecule (CADM) family contains four proteins in the immunoglobulin containing super family that are associated with cell-cell junctions^9^. The four members of the CADM family all share three extracellular immunoglobulin (Ig) repeats as well as a single transmembrane domain and a short cytosolic region on the C-terminus^10^. In addition to cell-cell junctions, CADMs are known to play a role in neurobiology^11–13^ and spermatogenesis^14^. CADM family members are generally regarded as tumor suppressors. For instance, CADM4 has been shown to suppress colon cancer tumorigenicity^15^, and CADM2 may play a tumor suppressive role in prostate cancer as epigenetic silencing and deletion of the *CADM2* locus has been frequently observed^16,17^. Similarly, CADM1 (also known as TSLC1, NECL-2, IGSF4, SynCAM1) serves as a tumor suppressor in a variety of human cancers including lung^18,19^, nasopharyngeal carcinoma^20^, among others (reviewed in^21^). CADM molecules function via either homophilic or heterophilic dimerization^22^. These interactions link to the actin cytoskeleton through recruitment of DAL-1/4.1B actin binding proteins as well as membrane-associated guanylate kinases (MAGuKs) as scaffolds^23–25^. Thus, CADM family proteins may be involved in cell-cell adherence and potentially play a role in EMT-like processes and metastatic progression.

Using melanoma as a model system, we demonstrate that CADM1 is a critical negative regulator of metastatic traits. CADM1 was found to be repressed by the transcription factor TWIST1. This repression persisted across multiple melanoma cell lines of different genetic backgrounds. We found that CADM1 expression in melanoma reduces migratory and invasive potential and potently induces cell death in non-adherent cells. Furthermore, high CADM1 expression in patient samples was linked to less aggressive melanomas and associated with improved progression free and overall survival. These findings highlight CADM1 as a possible prognostic marker.

## Results

### TWIST1 regulates expression of EMT and cell adhesion molecule pathways

**Fig. 1:**
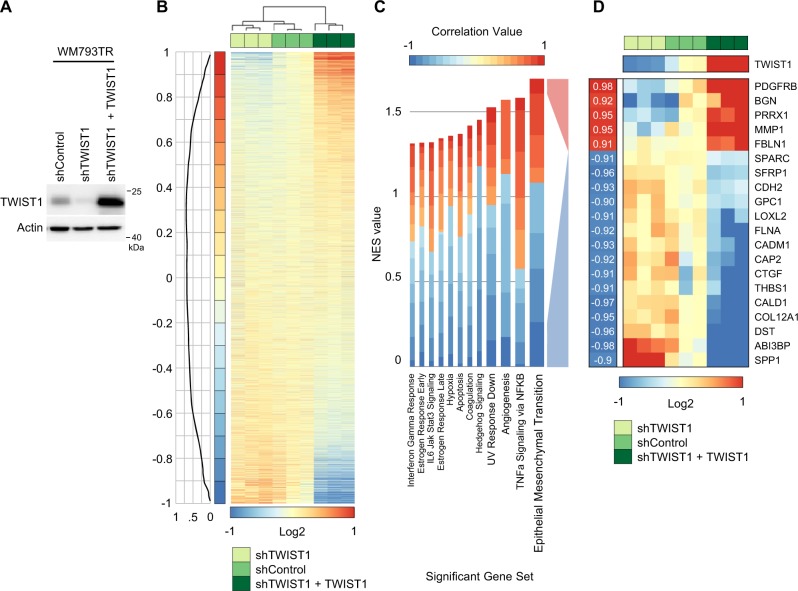
Microarray analysis of TWIST1 regulated genes. **a** Western blot analysis of WM793TR cells utilized in microarray analysis. **b** A heat map displaying median-centered log2 expression levels for each sample with the data on a correlation value interval scale. Above the sample numbers is a dendrogram plot displaying log-transformed linkage distances with samples arranged in optimal leaf order. Clusters are based on expression values for the top 250 genes positively or negatively correlating with TWIST1. A line plot showing kernel smoothing of probability density function estimates for gene correlation values is on the left, followed by the correlation value range. Sample conditions are displayed by different shades of green. **c** GSEA results showing statistically significant (BH FDR < 0.05) Hallmark gene sets. Normalized enrichment score (NES) values for each gene set are represented by the height of the bars. The color scale represents correlation values ranging from −1 to 1 with a color gradient interval rate of 0.1. Gene set NES bars are colored based on the fraction of enriched genes having a correlation value within a given range. The width of the bars represents the significance cutoff level, with FDR < 0.001, 0.01, 0.05 ranging from largest to smallest, respectively. **d** A heat map containing the EMT hallmark gene set genes (*n* = 20) with an absolute Pearson correlation value > 0.9. Genes are sorted based on hierarchical clustering. Pearson correlation r-values are displayed to the left of the indexing column

TWIST1 promotes EMT and metastatic-traits, but the repertoire of TWIST1 targets is not well understood^3–7^. We explored the TWIST1-regulated transcriptome through expression array analysis using invasive mutant BRAF melanoma cells as a model. Vertical growth phase (VGP) WM793TR cells expressing control shRNA, TWIST1 shRNA, or TWIST1 shRNA and a CMV-regulated TWIST1 rescue construct were assayed (Fig. 1a). Median-centered log_2_ expression values were represented via heatmap (Fig. 1b). Samples were ordered by optimal leaf ordering and a probability curve of each gene’s correlation to TWIST1 expression is provided (Fig. 1b left). Using geneset enrichment analysis (GSEA) to query mSigDB’s Hallmark Pathways, the highest scoring TWIST1-regulated pathway was Epithelial Mesenchymal Transition (EMT) (Fig. 1c). The most strongly correlated genes in the EMT pathway were further analyzed. Genes from the EMT hallmark pathway with an absolute Pearson correlation value of >0.9 were listed with the associated *r*-value and heatmap representation of expression (Fig. 1d). Taken together, these results suggest that TWIST1 regulates EMT pathway-related genes in melanoma.

### CADM1 is negatively regulated by TWIST1

**Fig. 2:**
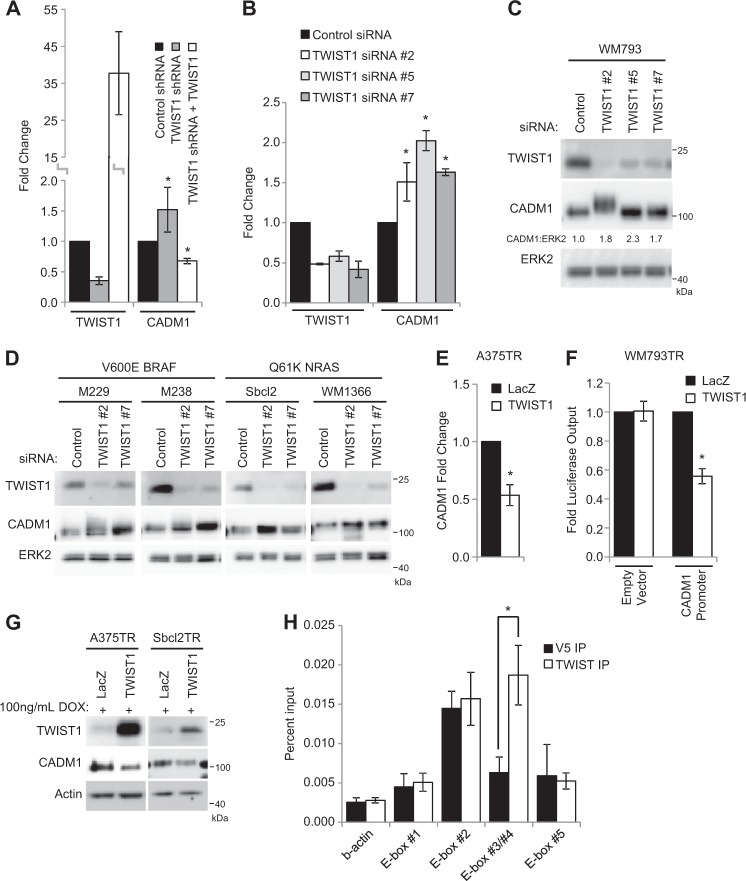
CADM1 is negatively regulated by TWIST1 in human melanoma cell lines. **a** Validation of microarray via qRT-PCR analysis. **b-c** qRT-PCR (**b**) and protein levels (**c**) of TWIST1 and CADM1 after control or TWIST1 siRNA transfections in WM793 cells. Densitometry for the ratio of CADM1 to ERK2 is indicated. **d** Lysates were probed via Western blotting after knockdown of TWIST1 levels by siRNA transfections in the mutant BRAF melanoma cell lines M229 and M238, and the mutant NRAS cell lines, Sbcl2 and WM1366. TWIST1 and CADM1 protein levels were assayed with ERK2 as a loading control. **e** qRT-PCR levels of CADM1 in A375TR cells after induction of either control LacZ or TWIST1. **f** Reporter constructs consisting of either an empty vector control or 1047bp upstream of CADM1 putative start site were transfected into WM793TR LacZ or WM793TR TWIST1 cells. Luciferase output was measured as an indication of promoter activity. Results are averages from three independent repeated experiments. Fold luciferase output is normalized to the levels of WM793TR LacZ cells per each luciferase construct. * represents *p* < 0.05 as determined by t-test, error bars are ±SEM. **g** CADM1 protein levels were measured via western blot after 96 h of exogenous TWIST1 expression. **h** Chromatin immunoprecipitation (ChIP) was performed on WM793 cells samples with TWIST1 antibody or V5 epitope antibody as a negative control. Associated genomic DNA was then subjected to qRT-PCR to determine the enrichment of each area of interest indicated in Supplementary Fig. 2. Results are averages from three independent repeated experiments. * represents *p* < 0.05 as determined by t-test, error bars are ±SEM

Analysis of the most highly regulated TWIST1 targets directed us to further investigate CADM1, due to its potential role as a tumor suppressor^21^. Microarray findings were validated by qRT-PCR, confirming that CADM1 is inversely associated with TWIST1 transcript level (Fig. 2a). CADM1 transcript and protein levels were increased after siRNA mediated knockdown of TWIST1 with multiple independent siRNAs (Fig. 2b, c). It is noteworthy that treatment with TWIST1 siRNA #2 caused a shift in the molecular weight of CADM1, an effect that may warrant further exploration. The inverse relationship of TWIST1 and CADM1 was consistent across cell lines regardless of mutant BRAF or NRAS genotype (Fig. 2d). Furthermore, inducible TWIST1 expression reduced CADM1 transcript levels (Fig. 2e), CADM1 promoter directed reporter activity (Fig. 2f), and suppressed CADM1 protein levels (Fig. 2g). TWIST1 has been reported to act as a transcriptional repressor for a number of genes including E-cadherin, ERα, and Sox9^26–28^. Five E-boxes, a well-defined TWIST1 binding motif, were identified in the ~ 1400 base pair *CADM1* proximal promoter region^3,29^ (Supplementary Fig. 1). This prompted us to investigate whether TWIST1 physically interacts with the E-boxes in *CADM1* promoter via chromatin immunoprecipitation (ChIP). ChIP of genomic extracts of WM793 cells demonstrated an enriched presence of TWIST1 at the *CADM1* promoter region containing E-box #3 and #4 (Fig. 2h). These data support the notion that TWIST1 is directly acting to repress CADM1 expression.

### Expression of CADM1 is inversely related to ERK1/2 signaling

**Fig. 3:**
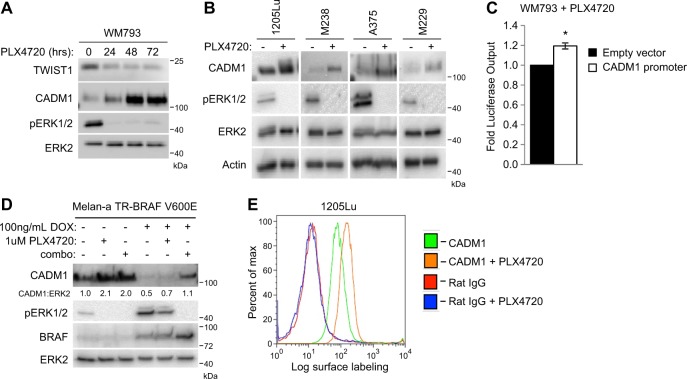
CADM1 levels are inversely related to ERK1/2 phosphorylation. **a** WM793 cells were treated with the RAF inhibitor, PLX4720, at 1 μM for the indicated time periods. Western blots for TWIST1, CADM1, phosphorylated ERK1/2 and total ERK2 were performed. **b** Lysates from the BRAF V600E cell lines 1205Lu, M238, A375 and M229 were harvested and probed via western blotting with the indicated antibodies after overnight treatment with 1 μM PLX4720. **c** WM793 cells were transfected with either control empty vector or CADM1 promoter luciferase expression vectors. Transfected cells were treated with DMSO or 1 μM PLX4720 for 24 h. Cell lysates were obtained and luciferase output measured via luminometer was normalized to DMSO treatment for each construct. Data are graphed from three independent experiments, * represents *p* < 0.05 as determined by *t*-test, and error bars are ±SEM. **d** An immortalized mouse melanocyte cell line, melan-a, transduced to express BRAF V600E under doxycycline control was subjected to overnight treatments of 1 μM PLX4720, combination of 1 μM dabrafenib and 16 nM trametinib, or DMSO vehicle, as indicated. Western blots for CADM1, phosphorylated ERK1/2, total ERK2 and BRAF were performed. **e** 1205Lu cells were treated with DMSO vehicle or 1 μM PLX4720 overnight and then prepared for cell surface labeling. Cells were probed with either a N-terminal CADM1 antibody (#ab138697) or normal Rat IgG, as a control. A GFP conjugated anti-rat secondary antibody was then used for detection of cell surface labeling via flow cytometry. Plots are representative of three independent experiments

Since TWIST1 levels are modulated by ERK1/2 signaling^7^, we tested the effects of ERK1/2 pathway inhibition on CADM1 expression. Western blot analysis of WM793 cells treated with the selective mutant BRAF inhibitor, PLX4720, (the tool-compound for vemurafenib^30^) revealed an increase of CADM1 associated with reduced phospho-ERK1/2 and TWIST1 levels (Fig. 3a). This response was consistent in all cell lines tested (Fig. 3b). CADM1 promoter activity was also increased after treatment with PLX4720 (Fig. 3c). Furthermore, this relationship was assayed in PLX4720-resistant cell lines^31^. Resistant cells treated with PLX4720 displayed a weak upregulation of CADM1 expression compared to parental cells (Supplementary Fig. 2A). To address if the link between CADM1 levels and ERK1/2 signaling is present in non-transformed melanocytes, we utilized the immortalized mouse melanocyte cell line, melan-a^32^. CADM1 levels in melan-a cells increased with suppression of phospho-ERK1/2. Additionally, CADM1 was decreased with inducible BRAF V600E expression, an effect that was reversed following BRAF-MEK pathway inhibition (Fig. 3d). RAF inhibitor alone had little effect possibly due to poor suppression of ERK1/2 signaling after mutant BRAF overexpression. In mutant BRAF melanoma cells, flow cytometry revealed that PLX4720 induced an increase of surface exposed CADM1 (Fig. 3e and Supplementary Fig. 2B). Taken together, these results suggest ERK1/2 inhibition is associated with reduction of TWIST1 and an increase of CADM1 levels.

### CADM1 expression reduces melanoma cell invasion and migration

**Fig. 4:**
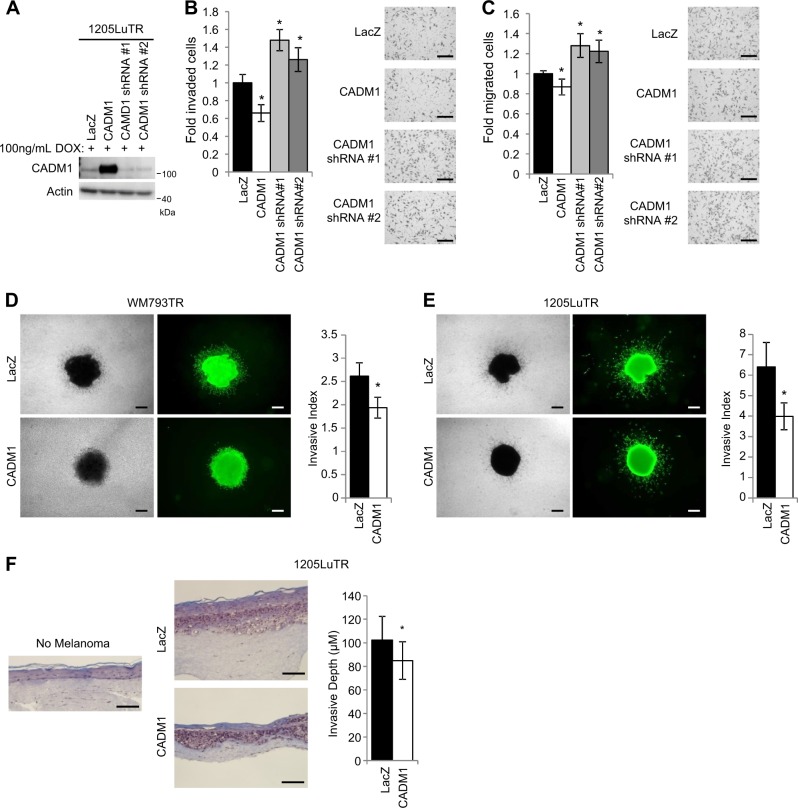
CADM1 decreases cellular motility and invasiveness. **a** Western blot analysis was used to validate CADM1 expression levels of doxycycline inducible 1205LuTR cells engineered to express control LacZ, CADM1, or CADM1 shRNAs. **b** Cells from (**a**) were subjected to matrigel-coated Boyden chamber invasion assays. Quantification of invaded cells, and representative images are shown. Data is graphed as a fold change compared to 1205LuTR LacZ invaded cells from at least three independent experiments, * represents *p* < 0.05 as determined by t-test, and error bars are ±SEM. Scale bar = 200 μM (**c**) Similar to (**b**), 1205LuTR cells were subjected to Boyden chamber based, serum directed migration assays. Scale bar = 200 μM (**d**, **e**) Spheroids of LacZ or CADM1 expressing WM793TR (**d**) and 1205LuTR (**e**) cells were assayed for their invasiveness into collagen. Representative images of phase contrast and green fluorescence via Calcien AM staining are shown. Invasive index is quantified from at least *N* = 40 per group across three different experiments. * represents p < 0.05 as determined by t-test, error bars are ±SEM. Scale bar = 200 μM. **f** Similar to (**d**) except that melanoma cell invasiveness was assayed in 3D skin reconstruct model. The depth of melanoma was measured from at least 70 measurements across two experiments. * represents *p* < 0.05 as determined by *t*-test, error bars are ±SEM. Scale bar = 200 μM

Since CADM1 is a cell adhesion protein, we tested if CADM1 could affect invasion and migration^7,33–37^. Human melanoma cell lines were transduced to inducibly express CADM1 or CADM1 shRNA constructs (Fig. 4a). In matrigel-coated Boyden chambers, CADM1 overexpression suppressed the invasiveness of melanoma cells, while CADM1 knockdown increased melanoma cell invasion (Fig. 4b). Similarly, in serum-directed migration assays, CADM1 overexpression reduced cell migration, while knockdown enhanced this process (Fig. 4c). Exogenous expression CADM1 elicited similar responses in WM793TR cells (Supplementary Figs. 3A-3B) and had a suppressive effect on 2D wound closure (Supplementary Fig. 3c). We further explored the effect of CADM1 expression on melanoma invasiveness by utilizing collagen-embedded 3D spheroid assays that models dermal outgrowth. Melanoma spheroids expressing CADM1 invaded significantly less than LacZ-expressing counterparts (Fig. 4d, e). These assays were expanded to a 3D human skin reconstruct model. Compared to LacZ expressing cells, CADM1 expression in 1205LuTR suppressed the invasive depth of the melanoma (Fig. 4f). CADM1 is also known to play a role in cell-cell junctions^38^, therefore, we visualized CADM1 localization and cell morphology. A375TR cells expressing CADM1 showed strong CADM1 localization to cell-cell boundaries and induced a cobblestone-like cell morphology (Supplementary Fig. 3D). Next, we tested the effect of BRAF inhibition on CADM1 localization. CADM1 was localized at the cell boundaries following PLX4720 treatment (Supplementary Fig. 3E). Additionally, both CADM1 expression and PLX4720 treatment resulted in cellular aggregation (Supplementary Figs. 3F and 3G). Together, these data demonstrate that CADM1 suppresses migratory and invasive potential, as well as induces cell adhesion and aggregation in human melanoma cells.

### CADM1 induces cell death in non-adherent conditions

**Fig. 5:**
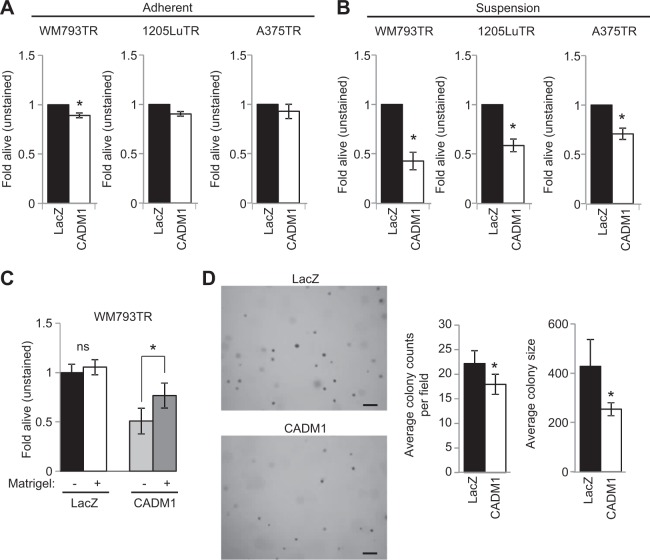
CADM1 induces cell death in non-adherent conditions. **a** WM793TR, 1205LuTR, and A375TR cells expressing LacZ or CADM1 were measured for cell death via annexin V/propidium iodine positivity. After overnight seeding, cell lines were treated with doxycycline to induce transgene expression for 24 h. Cells were harvested and assayed for annexin V/propidium iodine via flow cytometry. Data are represented as fold alive (unstained) compared to LacZ expression as a control from at least three independent experiments, * represents *p* < 0.05 as determined by *t*-test, and error bars are ±SEM. **b** Similar to (**a**) except that cells were seeded in bacto-agar coated wells. **c** WM793TR LacZ and CADM1 cells were seeded in bacto-agar coated wells with or without an injection of 80–100 μg of Matrigel. Data are represented as fold alive (unstained) compared to WM793 LacZ without Matrigel as a control from at least three independent experiments, * represents *p* < 0.05 as determined by t-test, and error bars are ±SEM. **d** Representative images after 21 days of colony growth for WM793TR LacZ or CADM1 cells in soft agar. Number of colonies per field and colony size are quantified. * represents *p* < 0.05 as determined by t-test, error bars are ±SEM. Scale bar = 200 μM

We next analyzed a role for CADM1 in resistance to anoikis is a key step to metastatic colonization of distant sites. Flow cytometry analysis of annexin V/propidium iodine staining indicated that while expression of CADM1 had either no to minor effects in adherent conditions (Fig. 5a), expression of CADM1 in non-adherent conditions strongly induced cell death (Fig. 5b). Levels of exogenous CADM1 were equivalent in adherent and non-adherent conditions within each cell line (Supplementary Fig. 4A). These effects were not general effects since expression of exogenous ErbB3, an alternative transmembrane protein, did not induce anoikis (Supplementary Figs. 4B, 4C). CADM1-induced cell death in non-adherent conditions was partially rescued when matrigel was added to cells in suspension, suggesting that the cell death effect of CADM1 is suppressed when cells are associated with extracellular matrix (Fig. 5c). It is noteworthy that while matrigel partially rescued the effects of CADM1 expression in suspension, the pan-caspase inhibitor, Z-VAD FMK, did not (Supplementary Figs. 4D-4F). Furthermore, while no changes in viability were observed for CADM1-expressing cells in 2D MTT assays, CADM1 expressing cells formed fewer and smaller colonies in soft agar growth assays (Supplementary Fig. 4G and Fig. 5d). Together these data implicate CADM1 as a suppressor of melanoma cell viability in non-adherent conditions.

### CADM1 expression in non-adherent conditions is associated with mitochondrial membrane depolarization and a parthanatos-like phenotype

**Fig. 6:**
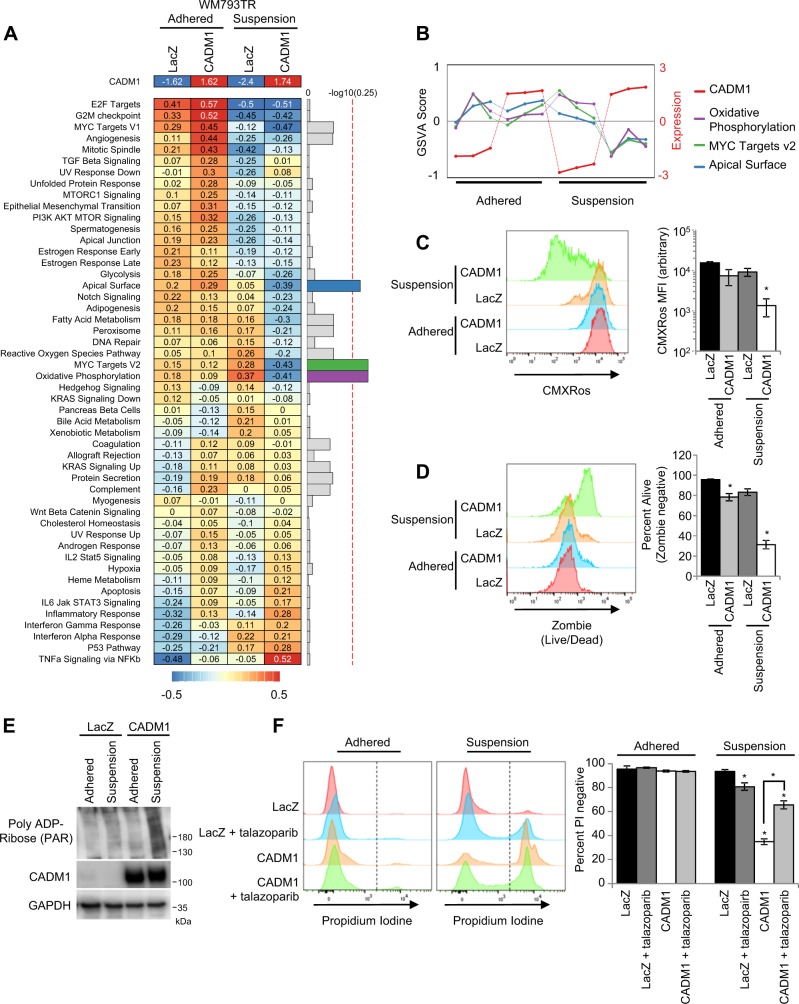
CADM1 expression in non-adherent conditions is associated with dysfunctional mitochondria and a parthanatos-like phenotype. **a** Heat map showing the median-centered log2 average CADM1 (top row) gene expression and MSigDB’s Hallmark gene set GSVA scores for each of the 2 condition factor groups (*n* = 3) (left panel). Gene sets were sorted based on hierarchical clustering of GSVA scores. Bar plot of the negative log10 transformed BH adjusted p-value for each gene set (right panel). The top 3 gene sets (all with BH adjusted *p*-values < 0.25) are arbitrarily colored while the rest are in gray. The red, dotted line represents BH adjusted *p*-value of 0.25. **b** Line plot of median-centered log2 CADM1 expression (red) and GSVA score values for CADM1 or LacZ expressing cell lines, plated or in suspension samples (n = 12) for the top 3 gene sets: Oxidative Phosphorylation (purple), MYC Targets v2 (green), and Apical Surface (blue). Colors correspond to those shown in part (**a**). **c** Representative stacked histogram and quantified average mean fluorescence intensity (MFI) of CMXRos stained LacZ or CADM1 expressing WM793TR cells in adherent or suspension conditions. * represents *p* < 0.05 as determined by 2-tailed t-test assuming unequal variance, and error bars are ±SEM. **d** Representative stacked histogram of Zombie staining and percent living cells as measured by absence of Zombie staining were quantified. Error bars and statistics are similar to (**c**). **e** Western blot analysis of poly ADP-Ribose (PAR) levels in whole cell extracts of LacZ or CADM1 expressing WM793TR cells cultured in the indicated conditions. **f** Representative stacked histograms of propidium iodine staining and quantified percentage of propidium iodine negative WM793TR LacZ and CADM1 cells in adhered and suspension conditions. Cells were treated with or without 5 μM talazoparib (PARPi)

CADM1 associated non-adherent cell death was not driven through caspase activity (Supplementary Fig. 4D-F), and was not a result of altered FAK, AKT, or BCL-2 family members (Supplementary Fig. 5A-B). Consequently, we performed microarray analysis to elucidate the differential roles of CADM1 in melanoma cells in adherent vs. non-adherent conditions. GSVA analysis identified three pathways (oxidative phosphorylation, myc targets V2, and apical surface) in the MSigDB Hallmark dataset associated with CADM1 expression in suspended cells (Fig. 6a). These three pathways have an interaction statistic of less than 0.25; an appropriate threshold indicated by GSEA for identification of target pathways. CADM1 expression and GSVA score of each of the twelve samples used in the study are shown (Fig. 6b). These three pathways demonstrated consistent GSVA scoring except for reduced scores when CADM1 is expressed in suspension.

We focused on oxidative phosphorylation since it has been linked to adhesion-related effects on cell survival in breast cancer^39^ and CADM1 plays a role in mitochondrial biology in human lung papillary adenocarcinoma^40^. We tested CADM1 effects on mitochondrial dysfunction by staining cells with Mitotracker GreenFM and CMXRos (stains for total mitochondria and mitochondrial membrane potential, respectively) followed by flow cytometry analysis. We observed a significant reduction in CMXRos staining, indicating poorly polarized mitochondrial membranes when CADM1-expressing cells were subjected to non-adherent conditions (Fig. 6c). Similar to the annexin V/propidium iodine based experiments, this reduction in membrane potential was associated with a significant decrease of living cells, as indicated by a multiplexed Zombie stain (Fig. 6d).

To identify how CADM1 expression in non-adherent conditions elicits cell death, we investigated mechanisms which coupled mitochondrial membrane depolarization with caspase-independent cell death. Hyperactivated poly(ADP-ribose) polymerase 1 (PARP1) plays a role in mitochondrial membrane depolarization and has been implicated as a mediator of caspase-independent cell death^41–43^. Western blotting demonstrated increased levels of poly ADP-ribose when CADM1 was expressed in non-adherent conditions (Fig. 6e). We then measured cell viability via propidium iodine staining in cells treated with or without the PARP inhibitor talazoparib (PARPi) in both adherent and non-adherent conditions. PARPi or CADM1 expression did not affect propidium iodine staining of adhered cells; although PARPi elicited a modest increase in cell death of LacZ-expressing cells in suspension (Fig. 6f). Interestingly, PARPi treatment rescued ~ 50% of the CADM1-induced non-adherent cell death (Fig. 6f). Similar results were obtained with a second PARPi, olaparib (Supplementary Fig. 5B). Taken together, these data suggest that CADM1 expression in non-adherent conditions leads to mitochondrial membrane depolarization and cell death, at least in part, via a mechanism involving PARP1.

### CADM1 is reduced in melanoma progression and is correlated with overall survival

**Fig. 7:**
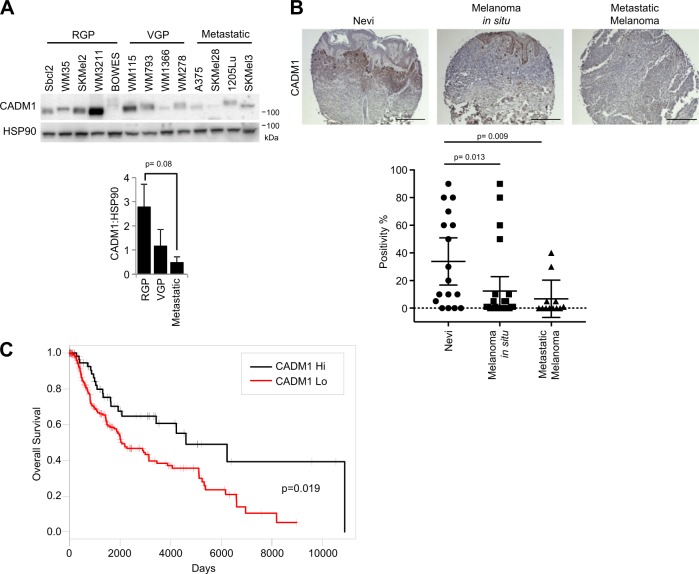
CADM1 is reduced in melanoma progression and is correlated with overall survival. **a** Western blots of a panel of melanoma cells grouped into radial growth phase (RGP), vertical growth phase (VGP), or metastatic. The ratio of CADM1 to HSP90 is graphed (lower). **b** Human melanoma tissue microarray slide was probed with CADM1 antibody. Representative images are shown. Percent positivity of each sample was scored by a blinded pathologist. Data for nevi (*n* = 17), in situ melanoma (*n* = 26) and metastatic melanoma (*n* = 12) samples are graphed and *p*-values comparing the groups are indicated. Scale bar = 200 μM. **c** Kaplan-Meier plot of overall survival (in days) of melanoma patients separated into Hi and Low CADM1 expression. Data downloaded from the Broad Institute (http://gdac.broadinstitute.org/)

Since CADM1 reduced migration and invasion, and elicited an anoikis-like phenotype in non-adherent conditions, we hypothesized that CADM1 would be expressed at lower levels in more aggressive tumors. A human melanoma panel containing radial growth phase, vertical growth phase (VGP), and metastatic cell lines were assayed for CADM1 expression (Fig. 7a). Western blotting revealed CADM1 levels were lowest in cell lines with more aggressive behavior. Similar results were obtained when we expanded these studies to a human melanoma tissue microarray. Immunohistochemical staining suggested that CADM1 decreases from nevi to melanoma in situ to metastatic melanoma (Fig. 7b). We next questioned whether CADM1 expression was associated with a better prognosis in melanoma patients. We utilized the publicly available SKCM TCGA dataset to analyze CADM1 expression as an indicator of overall survival (OS) and progression free survival (PFS). In univariate survival analysis, log-transformed CADM1 transcript levels were found to be a significant predictor of OS, with higher levels of CADM1 associated with longer survival (hazard ratio (HR) is 0.87, 95% CI: 0.77, 0.98; *p* = 0.027). Similarly, patients with higher levels of CADM1 are associated with longer progression-free survival (HR = 0.68 (95% CI: 0.49, 0.94; *p* = 0.019)). Kaplan–Meier curve estimates of OS in 248 patients with low CADM1 expression (CADM1 ≤ 1960) vs. 68 patients with high CADM1 expression (CADM1 > 1960) show a significant increase in overall survival for CADM1 high patients (Fig. 7c). Additionally, a parsimonious Cox model demonstrates CADM1 is a significant predictor of OS and is borderline significant (*p* = 0.061) predictor of PFS (Supplementary Figs. 6A and 6B). The HR for PFS (HR = 0.71, 95% CI: 0.50, 1.02) is more profound than the HR = 0.75 for the CADM1 effect on OS, however the *p* = 0.061 is likely the result of limited number of events^16^ in 95 patients available for PFS analysis. Together, these data demonstrate that CADM1 levels are decreased as melanoma progresses and high levels of CADM1 are associated with better clinical outcomes.

## Discussion

TWIST1 is a prototypical transcription factor involved in EMT^44^, is overexpressed in many tumor types including melanoma^45,46^, and enhances invasion of melanoma cells through the dermal layer^7^. In this study, expression array analysis was used to provide novel insights into the TWIST1-regulated transcriptome. We found TWIST1 has a strong (Pearson correlation > 0.9) inverse correlation with the expression pattern of cell adhesion molecule 1 (CADM1). CADM1 was inversely associated with TWIST1 across melanoma genotypes and TWIST1 occupied the proximal *CADM1* promoter. We showed that CADM1 suppresses invasive and migratory potential of melanoma cells, and potently induces cell death when melanoma cells are cultured in non-adherent conditions. Our results are likely impactful since TCGA analysis suggests patients with high CADM1-expressing tumors have better OS. Our data support that CADM1 is acting as a suppressor of metastatic-like traits in melanoma cells and that CADM1 expression is correlated with an increase of OS in melanoma patients.

The aggressiveness of melanoma is thought to be a result of gene expression alterations creating a condition of enhanced self-renewal and motility that is unresponsive to differentiation cues^47^. The tumor microenvironment and cell-cell interactions play a key role in tumor progression. As an adhesion molecule, CADM1 may be contributing to cellular junctions which propagate proliferative, motility, or differentiation signals. Interestingly, CADM1 has been implicated in the biology of de-differentiated cells. CADM1 expression reduced the oncogenic potential of hepatic cancer stem cells^48^ and inhibited the proliferation, migration, and wound healing abilities of the stem-cell like hair follicle bulge keratinocytes^49^. Our cell line panel and tissue microarray analysis may support these findings as CADM1 expression decreases in more aggressive cells. While the function of CADM1 in the formation of nevi is unknown, our data suggest CADM1 plays multiple tumor suppressive functions that would likely be selected against as nevi clonally evolve to in situ and metastatic disease.

We linked CADM1 expression to ERK1/2 activity. Specifically, when cells were treated with inhibitors to suppress phosphoERK1/2 levels, TWIST1 levels were also decreased and CADM1 increased. Thus, CADM1 may represent a new target of RAS-RAF-MEK-ERK1/2 pathway inhibition in metastatic melanoma patients. Augmented CADM1 expression significantly increased cell death in non-adherent conditions, and reduced the invasive and migratory potential of melanoma cells. These data suggest that the increase of CADM1 resulting from treatment with BRAF and MEK inhibitors may suppress the ability of tumor cells to intravasate safely into the bloodstream and may, at least in part, underlie the observation that patients on ERK1/2 pathway suppression treatment have less circulating tumor cells^50^. In addition, a recent phase 2 trial found that Stage III melanoma patients treated with BRAFi/MEKi as a neoadjuvant prior to surgery and as adjuvant after surgery, had a significantly improved event-free survival compared to standard of care^51^. Perhaps CADM1 upregulation resulting from RAF-MEK-ERK pathway inhibitors is contributing to the favorable outcomes in this trial.

We found that CADM1 expression in non-adherent conditions leads to cell death. This observation may highlight a role for CADM1 in metastatic progression by acting as a mediator of for anoikis resistance. Notably, endogenous levels of CADM1 were reduced when melanoma cell lines were cultured in non-adherent conditions (Supplementary Figs. 4A-4B). This response may represent a survival mechanism employed by circulating tumor cells. Interestingly, CADM1-induced non-adherent cell death was apparently not driven through caspase-driven pathways. We found that when CADM1 is expressed in non-adherent conditions, cell death was associated with decreases in mitochondrial membrane potential and an increase of poly-ADP ribose staining. Caspase-independent cell death resulting from increased PARP activity is termed parthanatos^52,53^ and was recently implicated in melanoma resistance to targeted inhibitors^54^. Given the high mutational load of melanoma, PARP inhibitors (PARPi) were thought to represent an efficacious treatment avenue; however, clinical trials with PARPi in melanoma patients have yielded poor results^55^. Consistent with the potential susceptibility to CADM1-induced non-adherent cell death, PARPi treatment produces differential effects on circulating tumor cells and a PARPi clinical trial of castration-resistant prostate cancer found a subset of patients with increased levels of circulating tumor cells after treatment^56^. Our present study indicating PARPi suppression of CADM1-induced non-adherent cell death provides a rationale for these clinical findings and could be a factor in PARPi efficacy.

The work presented here demonstrates a previously unknown function of TWIST1 in the metastatic progression of human melanoma. We found that TWIST1 acts to repress CADM1 levels and suppression of CADM1 appears to enhance traits needed for metastatic dissemination of melanoma. In melanoma disease progression, CADM1 may act as a barrier to metastasis by inhibiting the invasiveness, motility, and inducing cell death in non-adherent conditions. While further studies are needed to address the role of CADM1 in early steps of melanomagenesis, CADM1 expression in late stage disease may correlate with prognosis.

## Methods

### Cell culture

WM793, 1205Lu, WM1366, and Scbl2 cells (all donated by Dr. Meenhard Herlyn, Wistar Institute, Philadelphia, PA in 2005) were cultured in 37 °C humidified chamber with 5% CO_2_ in MCDB 153 media containing 20% Leibovitz-L15 media, 2% fetal bovine serum, 0.2% sodium bicarbonate, and 5 μg/mL insulin. A375 cells (purchased from ATCC in 2005) were grown in DMEM with 10% FBS. M229 and M238 (donated by Dr. Antoni Ribas in 2010) cells were grown in RPMI + 10% FBS with 1% L-glutamine. Melan-a cells (donated by Dr. Dorothy Bennett in 2010) were grown in RPMI 1640 with 10% FBS, 1% L-glutamine, and 200 nM TPA. All media have 1% penicillin/streptomycin. Cells are routinely assayed for mycoplasma contamination with MycoScope kit (Genlantis, San Diego, CA). Cells were most recently assayed May of 2018. Cell line authentication via STR analysis was completed in April 2018 for WM793 and 1205Lu cell lines. A375 and WM1366 cells were confirmed by STR analysis in April and May of 2015. STR analysis for M238 and M229 cells was performed in April 2015 and November 2015, respectively, producing unique profiles, while all other cells matched known profiles.

### Chromatin immunoprecipitation

ChIP was performed as previously described^7^. Briefly WM793 lysates were sonicated, precleared with protein A/G agarose beads, then incubated with Protein G Dynabeads (Invitrogen) with either TWIST1 (Santa Cruz Biotechnology) or anti-V5 epitope antibodies (Invitrogen). Experiment was repeated four times. Primer pair sequences were: β-actin F: AGTGTGGTCCTGCGACTTCTAAG, R: CCTGGGCTTGAGAGGTAGAGTGT, CADM1 E-Box #1 F: TCCAGCGCATGTCATTAG, R: CCTGTGGGGATCAGTGC, CADM1 E-Box #2 F: GATCTATTCCTTCGGTGCAAGG, R: CGGGTCTAGCTTCTTGTACACC, CADM1 E-Box #3/4 F: AGAGACAGGGGAAGCTCG, R: TTAGTCAAGGCTTCAGGTGAAGAGC, CADM1 E-Box #5 F: GTCGGCAGGATTGCTTATTTC, R: GCTTTCTTTTCAGCCAGGC.

### Statistics

Unless specified, all experiments were independently performed three times. Error bars represent + /− SEM. Significance was determined using a 2-tailed student’s *T*-test assuming unequal variance. *p* ≤ 0.05 was considered significant and represented with a *. For the GSVA analysis of the CADM1 microarray dataset, the interaction statistic is defined by a change that cannot be explained by 2D vs. suspension alone, or by LacZ vs. CADM1 expression alone, rather the interaction of both of these conditions are considered simultaneously for analysis.

### Annexin V/Propidium iodine staining

2 × 10^5^ (adherent) or 3 × 10^5^ (non-adherent) cells were seeded on 6-well dishes without or with a pre-coat of Bacto agar. The next day, doxycycline was added at 100 ng/mL to induce transgene or shRNA expression. After 24 h, cells were harvested an incubated with 5 uL of Annexin V-APC (BD Biosciences) and 0.2 mg/mL propidium iodine in 200 uL of 1x binding buffer (140 mM NaCl, 2.5 mM CaCl_2_, and 10 mM HEPES) for 15 min. Cells were then analyzed on FACS Calibur Flow Cytometer.

### Mitochondrial membrane potential assays

WM793TR LacZ and CADM1 cells were seeded in 6-well plates, or ultra-low adherent 6-well plates. The next day, cells were dosed with doxycycline at 100 ng/mL to induce transgene expression. Cells were incubated for ~20 h, collected, and stained for 40 min at 37 °C with Mitotracker Red CMXRos (Invitrogen #M7512) at 100 nM and Mitotracker GreenFM (Invitrogen #M7514) at 100 nM. Cells were then washed 2x with PBS and stained with Zombie Aqua (Biolegend #423101) (0.2 uL per 100 uL PBS) for 10 min. Samples were then washed and resuspended in FACS buffer (1% FBS and 0.05% sodium azide in ×1 PBS) for analysis on BD Celesta. Experiments were independently performed 3 times and data was analyzed with FlowJo software. Debris was gated out via FSC/SSC and then only Mitotracker GreenFM positive cells were used to assay CMXRos.

### Skin reconstructs

Skin reconstructs were created similar to^57^. 1205LuTR LacZ and CADM1 cells (1 × 10^5^) were added to the epidermal layer for analysis. Skin reconstruct media is changed every two days. After seven days of incubation, skins were dosed with DMSO or doxycycline at 100 ng/mL and incubated an additional 7 days. Skins were fixed in 4% buffered formalin and paraffin embedding.

### Tissue microarray analysis

Tissue microarray (US Biomax #ME1004d) was stained with anti-CADM1 antibody (Abcam #ab138697). The percent positivity of tumor tissue was scored by a blinded pathologist. The scoring of non-pigmented lesions was then categorized into cohorts of benign skin nevi, in situ melanoma, and metastatic melanoma. Statistics were analyzed with the independent samples Kruskal-Wallis test using Bonferroni corrections for multiple comparisons in Graphpad Prism.

### Soft agar colony formation

Briefly, 1 mL of 0.8% bacto-agar in WM media used to coat the bottom of a 12-well plate. After solidifying, 2 mL of 0.5% bacto-agar in WM media with 4000 cells/mL was overlaid. Finally, 1 mL of WM media with doxycycline (100 ng/mL final concentration) was added. Medium was routinely changed, and images were taken after four weeks of incubation with a Nikon Eclipse Ti inverted microscope (Nikon, Melville, NY) with NIS-Elements AR 3.00 software (Nikon) equipped with 4x objective. Three random fields per well were documented, WM793TR LacZ cells and CADM1 cells were seeded in triplicate for each experiment. Experiment was repeated four times. Images were quantified by ImageJ software.

### siRNA transfection

Cells were transfected for 4 h with siRNAs (Dharmacon) at a final concentration of 25 nM using oligofectamine (Invitrogen). Cells were harvested for lysates 72 h after transfections. TWIST1 siRNA sequences: #2 CUGCAG ACGCAGCGGGUCA, #5 GGAGUCCGCAGUCUUAC GA, #7 UGAGCAACAGCGAGGAAGA.

### Quantitative reverse transcriptase-PCR

RNA was extracted using PerfectPure RNA Cultured Cell Kit (5Prime) as per the manufacturer's instructions. RNA was then converted to cDNA using iScript cDNA Sythesis Kit (Bio-Rad). Analysis was done by utilizing iQ SYBR Green Supermix (Bio-Rad) in quantitative reverse transcriptase qRT-PCR. Relative fold changes in mRNA levels were calculated after normalization to β-actin using the comparative *C*_t_ method^58^. qRT-PCR primers: CADM1 F: TTTGAAGGACAGCAGGTTTCA, CADM1 R: AGGACTGTGATGGTGGTGTAACT, TWIST1 F: AGTCTTACGAGGAGCTGCAGACG, TWIST1 R: AGGAAGTCGATGTACCTGGCCG

### Inhibitors

Z-VAD FMK, PLX4720, trametinib, dabrafenib, talazoparib, and olaparib were purchased from Selleck (Houston, TX).

### Western blotting

Blotting was performed as previously described^59^. Antibodies used were: TWIST1 (#sc-81417), ERK2 (#sc-1647), and BRAF (#sc-166) from Santa Cruz Biotechnology (Dallas, TX), CADM1 antibodies #ab3910 and #ab138697 were purchased from Abcam (Cambridge, MA). pERK1/2 (#9101), Cleaved Caspase 3 (#9661), ErbB3 (#4754), and GAPDH (#2118) was purchased from Cell Signaling (Danvers, MA). Actin (#A2066) was from Sigma (St. Louis, MO) and Poly(ADP-ribose) (PAR) (#ALX-804-220-R100) was purchased from Enzo (Farmingdale, NY). Anti-V5 epitope was purchased from Invitrogen (Carlsbad, CA). Chemiluminence was visualized with VersaDoc MultiImager and quantified using Quantity-One software (Bio-Rad, Hercules, CA).

### Microscopy

7 × 10^4^ cells were cultured on glass coverslips overnight, and then washed with PBS, and fixed in ice cold methanol. Fixed cells were then stained overnight at 4 °C with anti CADM1 (Abcam #3910) at 1:200. Primary antibody was washed, then stained with an anti-rabbit GFP conjugated secondary antibody (Molecular Probes, Grand Island, NY) at 1:2000. Secondary antibody was then washed and cells were counter stained with TRITC conjugated phalloidin (Sigma, St. Louis, MO) and mounted with Prolong Gold Antifade reagent with DAPI (Molecular Probes, Grand Island, NY). Slides were imaged on Nikon Eclipse Ti inverted microscope (Nikon, Melville, NY) with NIS-Elements AR 3.00 software (Nikon) utilizing a 40x objective.

### Invasion and migration assay

Cells were treated with doxycycline at 100 ng/mL to induce expression of LacZ, CADM1, or shRNA constructs. After 24 h, cells were serum starved for an additional 24 h. For invasion assays, 8.0 μm cell culture inserts from BD Biosciences (San Jose, CA) were coated with 0.75 mg/ml Matrigel; migrations assays used non-coated 8.0 μM boyden chambers. Serum starved cells (3.75 × 10^4^) were seeded in the chamber with serum-free media supplemented with doxycycline. Cells were left to migrate or invade though Matrigel towards serum containing media attractant for a total of 24 h. Inserts were then removed, and fixed with buffered formalin. Cells were then stained with crystal violet, and membrane inserts were mounted for microscopic analysis. Images were taken using 10x objective lens on the Nikon Eclipse Ti inverted microscope (Nikon, Melville, NY). A total of 3 random fields were analyzed for each membrane. Quantification was performed utilizing NIS Elements (Nikon). The experiment was carried out three independent times in duplicate (for a total of 18 cell counts per experimental condition).

### Spheroid outgrowth assay

96-well plates were coated with 50 uL of 1.5% agarose in PBS. Cells (5 × 10^4^) were then seeded and allowed to form spheroids for 5–7 days. Spheroids were collected with a transfer pipette and resuspended in collagen mixture (per mL: 100 uL 10x reconstruct buffer (NaOH 0.05 M, NaHCO_3_ 2.2%, HEPES 200 mM), 100 uL HAM-F12 10x (Gibco #21700–26), 750 uL Collagen I Rat tail (Corning #354236) and 50 uL FBS). Collagen was left to solidify for ~two hours then WM media with doxycycline (final concentration of 100 ng/mL) was overlaid. After 48 h incubation, WM media was aspirated, and cells were then labeled by adding 300 uL of PBS containing 150 nM Calcien AM. Spheroids were then imaged by immunofluorescent microscopy using 10x objective. Invasive index was calculated by the area ratios of the sprouting cells per the area of the spheroid as determined by ImageJ. Each experimental group consisted of at least *n* = 40 across three different experiments.

### TCGA analysis

Cox proportional hazards model was used to model the OS and PFS with proportional hazards assumption validated. The following variables were considered as predictors of OS in the multivariate model: age_at_initial_pathologic_diagnosis, gender, tumor_weight, breslow_depth_value, history_of_neoadjuvant_treatment, sample_type, melanoma_ulceration_indicator, postoperative_rx_tx, radiation_therapy, BRAF V600 mutation, and NRAS Q61 mutation, as well as interactions between CADM1 levels and sample_type, BRAF V600 mutation, and NRAS Q61 mutation. In addition, the multivariate Cox model included covariates adjusting for TNM-staging. The variables pathologic_T, pathologic_N, and pathologic_M were grouped into categories 0–4 according to the numerical status with resulting covariates T-stage, N-stage, and M-stage. Due to a small number of patients with Stage M1, the M-stage was not used in multivariate Cox models. Also, 23 patients with T-Stage = 0 and melanoma ulceration indicator missing were excluded from the multivariate analysis of OS. The final Cox model for OS was obtained by backward elimination of non-significant predictors of OS. Since the PFS data available for analysis includes only 17 events in 114 patients, each stage variable (T-stage, N-stage) and other covariate was considered with CADM1 in separate Cox models. The data were analyzed in R (The R Foundation for Statistical Computing http://www.R-project.org).

### Scratch wound assay

1205LuTR LacZ or CADM1 cells (3.75 × 10^5^) were seeded in 6-well dishes. The next day cells were treated with 100 ng/mL doxycycline to induce transgene expression. Cells were then grown to confluence (~4 days), and which point a 2 mL pipette tip was used to scratch a “wound” in the confluent monolayer. Images were taken with 4x objective for T = 0. 4× images also taken at T = 48 h. Length of each wound was determined with NIS Elements (Nikon) at 5 different points along the scratch. Data is represented as fold wound opening from T = 0. Experiments were performed three times.

## Supplementary information

Supplemental Data
