# Supplementary material for: CADM1 is a TWIST1-regulated suppressor of invasion and survival
Source: Cell Death Dis. 2019 Mar 25;10(4):281. doi: 10.1038/s41419-019-1515-3 (PMC6433918; doi:10.1038/s41419-019-1515-3)
Supplement: Supplementary file 1 — Supplemental Data [file 41419_2019_1515_MOESM1_ESM.ppt]

## Slide 1
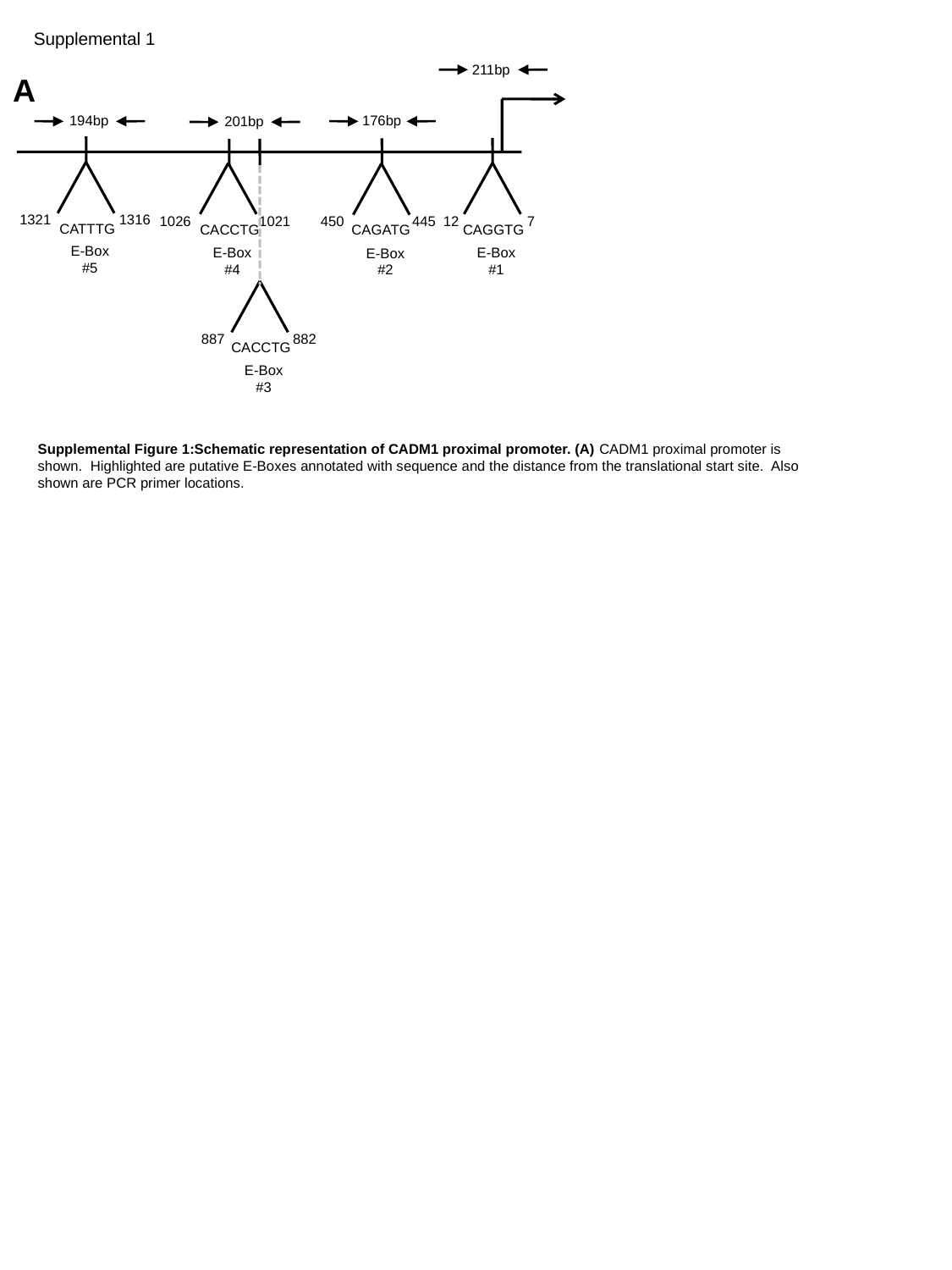

Supplemental 1
211bp
A
194bp
176bp
201bp
1321 1316
12 7
1026 1021
450 445
CATTTG
CACCTG
CAGGTG
CAGATG
E-Box
#5
E-Box
#4
E-Box
#1
E-Box
#2
887 882
CACCTG
E-Box
#3
Supplemental Figure 1:Schematic representation of CADM1 proximal promoter. (A) CADM1 proximal promoter is shown. Highlighted are putative E-Boxes annotated with sequence and the distance from the translational start site. Also shown are PCR primer locations.

## Slide 2
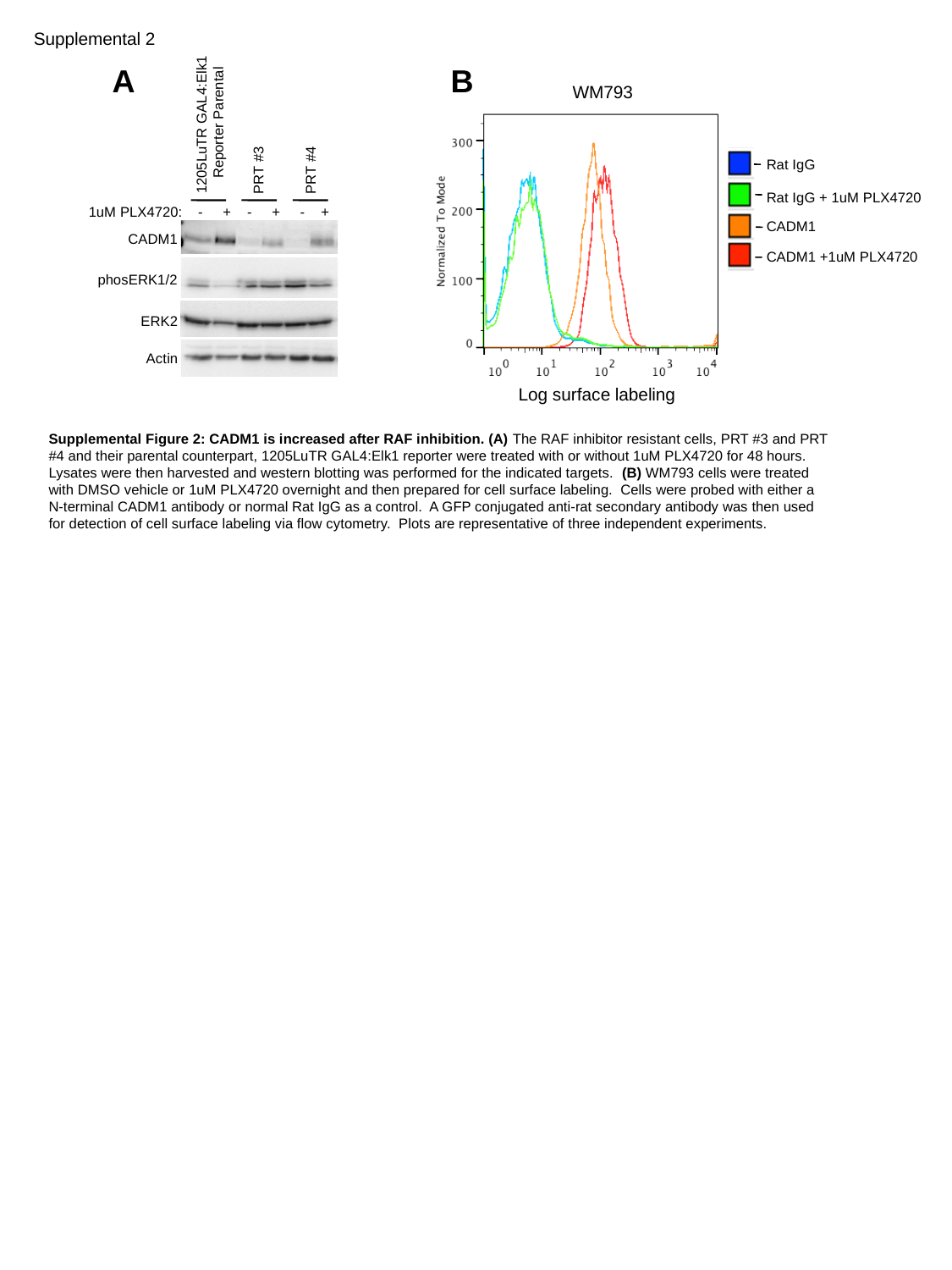

Supplemental 2
A
B
WM793
1205LuTR GAL4:Elk1
Reporter Parental
Rat IgG
PRT #3
PRT #4
Rat IgG + 1uM PLX4720
1uM PLX4720: - + - + - +
CADM1
CADM1
CADM1 +1uM PLX4720
phosERK1/2
ERK2
Actin
Log surface labeling
Supplemental Figure 2: CADM1 is increased after RAF inhibition. (A) The RAF inhibitor resistant cells, PRT #3 and PRT #4 and their parental counterpart, 1205LuTR GAL4:Elk1 reporter were treated with or without 1uM PLX4720 for 48 hours. Lysates were then harvested and western blotting was performed for the indicated targets. (B) WM793 cells were treated with DMSO vehicle or 1uM PLX4720 overnight and then prepared for cell surface labeling. Cells were probed with either a N-terminal CADM1 antibody or normal Rat IgG as a control. A GFP conjugated anti-rat secondary antibody was then used for detection of cell surface labeling via flow cytometry. Plots are representative of three independent experiments.

## Slide 3
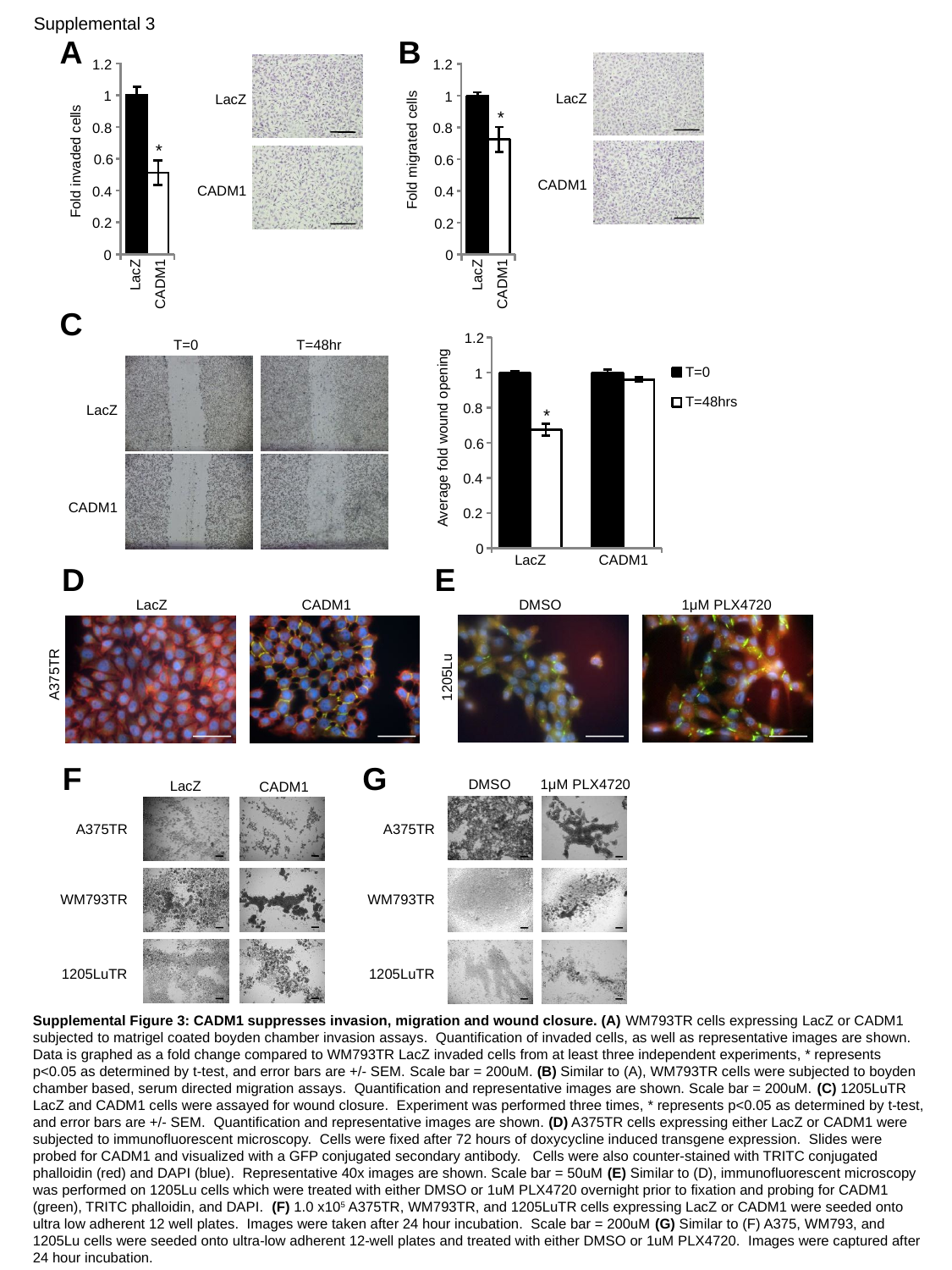

Supplemental 3
A
B
1.2
1.2
LacZ
LacZ
1
1
*
0.8
0.8
*
Fold migrated cells
Fold invaded cells
0.6
0.6
CADM1
CADM1
0.4
0.4
0.2
0.2
0
0
LacZ
LacZ
CADM1
CADM1
C
1.2
T=0
T=48hr
T=0
1
T=48hrs
LacZ
*
0.8
Average fold wound opening
0.6
0.4
CADM1
0.2
0
LacZ
CADM1
D
E
LacZ
CADM1
DMSO
1μM PLX4720
A375TR
1205Lu
F
G
DMSO
1μM PLX4720
LacZ
CADM1
A375TR
A375TR
WM793TR
WM793TR
1205LuTR
1205LuTR
Supplemental Figure 3: CADM1 suppresses invasion, migration and wound closure. (A) WM793TR cells expressing LacZ or CADM1 subjected to matrigel coated boyden chamber invasion assays. Quantification of invaded cells, as well as representative images are shown. Data is graphed as a fold change compared to WM793TR LacZ invaded cells from at least three independent experiments, * represents p<0.05 as determined by t-test, and error bars are +/- SEM. Scale bar = 200uM. (B) Similar to (A), WM793TR cells were subjected to boyden chamber based, serum directed migration assays. Quantification and representative images are shown. Scale bar = 200uM. (C) 1205LuTR LacZ and CADM1 cells were assayed for wound closure. Experiment was performed three times, * represents p<0.05 as determined by t-test, and error bars are +/- SEM. Quantification and representative images are shown. (D) A375TR cells expressing either LacZ or CADM1 were subjected to immunofluorescent microscopy. Cells were fixed after 72 hours of doxycycline induced transgene expression. Slides were probed for CADM1 and visualized with a GFP conjugated secondary antibody. Cells were also counter-stained with TRITC conjugated phalloidin (red) and DAPI (blue). Representative 40x images are shown. Scale bar = 50uM (E) Similar to (D), immunofluorescent microscopy was performed on 1205Lu cells which were treated with either DMSO or 1uM PLX4720 overnight prior to fixation and probing for CADM1 (green), TRITC phalloidin, and DAPI. (F) 1.0 x10^5^ A375TR, WM793TR, and 1205LuTR cells expressing LacZ or CADM1 were seeded onto ultra low adherent 12 well plates. Images were taken after 24 hour incubation. Scale bar = 200uM (G) Similar to (F) A375, WM793, and 1205Lu cells were seeded onto ultra-low adherent 12-well plates and treated with either DMSO or 1uM PLX4720. Images were captured after 24 hour incubation.

## Slide 4
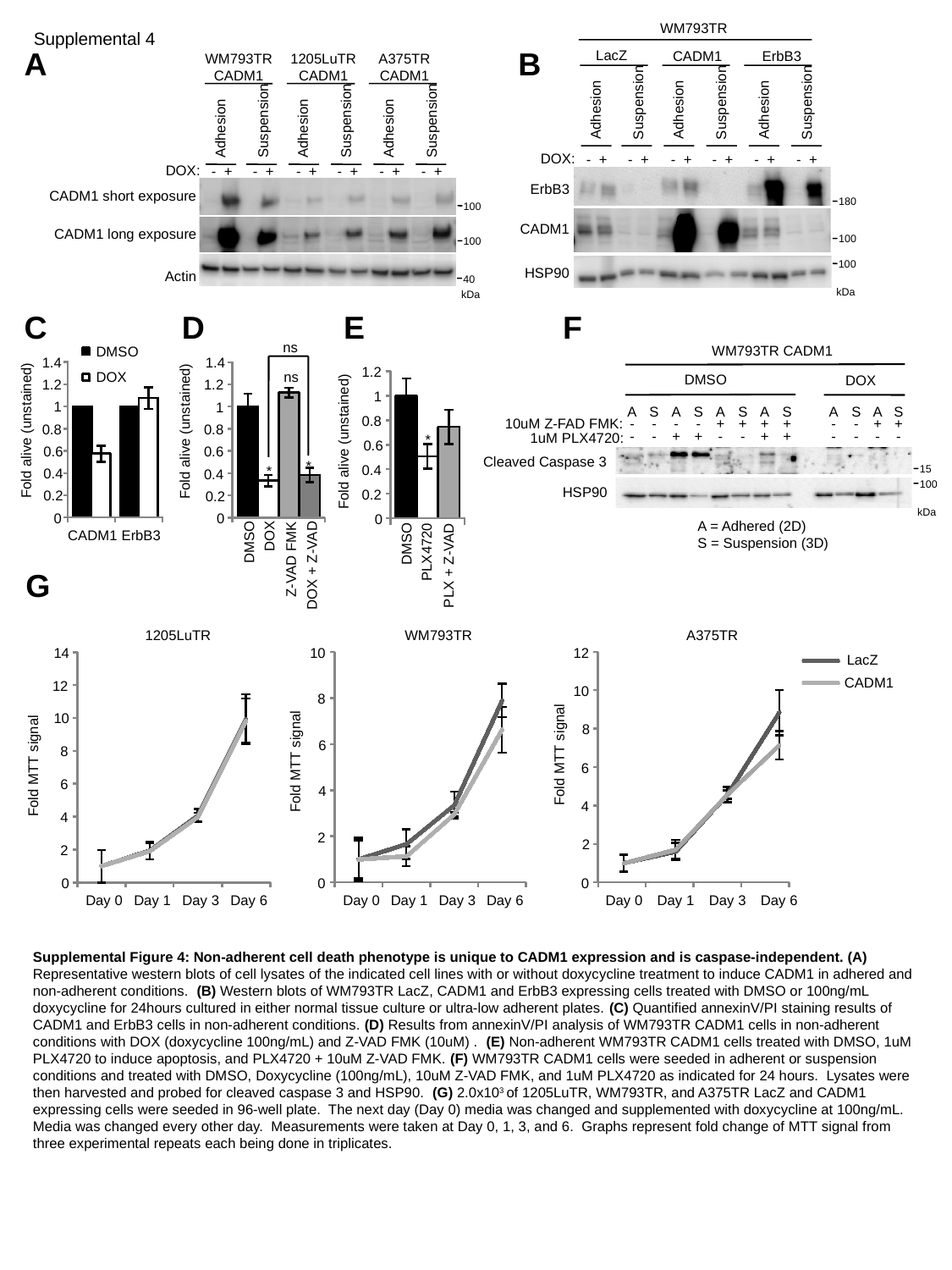

WM793TR
Supplemental 4
A
B
LacZ
CADM1
ErbB3
WM793TR
CADM1
1205LuTR
CADM1
A375TR
CADM1
Suspension
Suspension
Suspension
Adhesion
Adhesion
Adhesion
Suspension
Suspension
Suspension
Adhesion
Adhesion
Adhesion
DOX:
- +
- +
- +
- +
- +
- +
DOX:
- +
- +
- +
- +
- +
- +
ErbB3
CADM1 short exposure
180
100
CADM1
CADM1 long exposure
100
100
100
HSP90
Actin
40
kDa
kDa
C
D
E
F
WM793TR CADM1
DMSO
ns
1.4
1.4
DOX
1.2
DMSO
DOX
ns
1.2
1.2
1
A
-
-
S
-
-
A
-
+
S
-
+
A
+
-
S
+
-
A
+
+
S
+
+
A
-
-
S
-
-
A
+
-
S
+
-
1
1
10uM Z-FAD FMK:
0.8
Fold alive (unstained)
Fold alive (unstained)
0.8
0.8
1uM PLX4720:
Fold alive (unstained)
*
0.6
0.6
0.6
Cleaved Caspase 3
15
*
0.4
*
0.4
0.4
100
HSP90
0.2
0.2
0.2
kDa
0
0
0
A = Adhered (2D)
S = Suspension (3D)
CADM1
ErbB3
DOX
DMSO
DMSO
PLX4720
Z-VAD FMK
DOX + Z-VAD
PLX + Z-VAD
G
1205LuTR
WM793TR
A375TR
14
10
12
LacZ
CADM1
12
10
8
10
8
6
Fold MTT signal
8
Fold MTT signal
Fold MTT signal
6
6
4
4
4
2
2
2
0
0
0
Day 0
Day 1
Day 3
Day 6
Day 0
Day 1
Day 3
Day 6
Day 0
Day 1
Day 3
Day 6
Supplemental Figure 4: Non-adherent cell death phenotype is unique to CADM1 expression and is caspase-independent. (A) Representative western blots of cell lysates of the indicated cell lines with or without doxycycline treatment to induce CADM1 in adhered and non-adherent conditions. (B) Western blots of WM793TR LacZ, CADM1 and ErbB3 expressing cells treated with DMSO or 100ng/mL doxycycline for 24hours cultured in either normal tissue culture or ultra-low adherent plates. (C) Quantified annexinV/PI staining results of CADM1 and ErbB3 cells in non-adherent conditions. (D) Results from annexinV/PI analysis of WM793TR CADM1 cells in non-adherent conditions with DOX (doxycycline 100ng/mL) and Z-VAD FMK (10uM) . (E) Non-adherent WM793TR CADM1 cells treated with DMSO, 1uM PLX4720 to induce apoptosis, and PLX4720 + 10uM Z-VAD FMK. (F) WM793TR CADM1 cells were seeded in adherent or suspension conditions and treated with DMSO, Doxycycline (100ng/mL), 10uM Z-VAD FMK, and 1uM PLX4720 as indicated for 24 hours. Lysates were then harvested and probed for cleaved caspase 3 and HSP90. (G) 2.0x10^3^ of 1205LuTR, WM793TR, and A375TR LacZ and CADM1 expressing cells were seeded in 96-well plate. The next day (Day 0) media was changed and supplemented with doxycycline at 100ng/mL. Media was changed every other day. Measurements were taken at Day 0, 1, 3, and 6. Graphs represent fold change of MTT signal from three experimental repeats each being done in triplicates.

## Slide 5
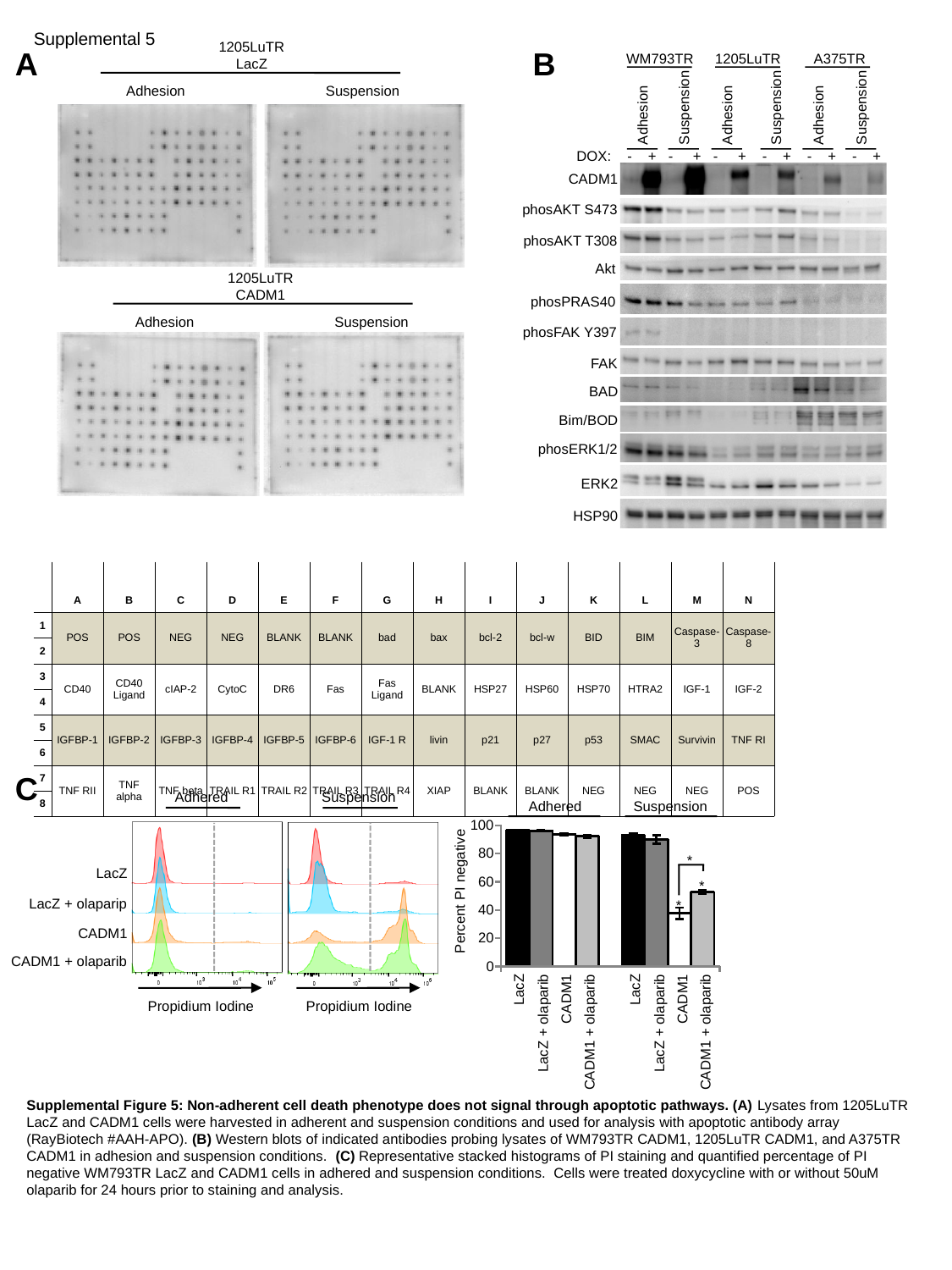

Supplemental 5
1205LuTR
LacZ
A
B
WM793TR
1205LuTR
A375TR
Adhesion
Suspension
Suspension
Suspension
Suspension
Adhesion
Adhesion
Adhesion
DOX: - + - + - + - + - + - +
CADM1
phosAKT S473
phosAKT T308
Akt
1205LuTR
CADM1
phosPRAS40
Adhesion
Suspension
phosFAK Y397
FAK
BAD
Bim/BOD
phosERK1/2
ERK2
HSP90
| | A | B | C | D | E | F | G | H | I | J | K | L | M | N |
| --- | --- | --- | --- | --- | --- | --- | --- | --- | --- | --- | --- | --- | --- | --- |
| 1 | POS | POS | NEG | NEG | BLANK | BLANK | bad | bax | bcl-2 | bcl-w | BID | BIM | Caspase-3 | Caspase-8 |
| 2 | | | | | | | | | | | | | | |
| 3 | CD40 | CD40 Ligand | cIAP-2 | CytoC | DR6 | Fas | Fas Ligand | BLANK | HSP27 | HSP60 | HSP70 | HTRA2 | IGF-1 | IGF-2 |
| 4 | | | | | | | | | | | | | | |
| 5 | IGFBP-1 | IGFBP-2 | IGFBP-3 | IGFBP-4 | IGFBP-5 | IGFBP-6 | IGF-1 R | livin | p21 | p27 | p53 | SMAC | Survivin | TNF RI |
| 6 | | | | | | | | | | | | | | |
| 7 | TNF RII | TNF alpha | TNF beta | TRAIL R1 | TRAIL R2 | TRAIL R3 | TRAIL R4 | XIAP | BLANK | BLANK | NEG | NEG | NEG | POS |
| 8 | | | | | | | | | | | | | | |
C
Adhered
Suspension
Adhered
Suspension
100
80
*
LacZ
60
Percent PI negative
*
LacZ + olaparip
*
40
CADM1
20
CADM1 + olaparib
0
LacZ
LacZ
CADM1
CADM1
Propidium Iodine
Propidium Iodine
LacZ + olaparib
LacZ + olaparib
CADM1 + olaparib
CADM1 + olaparib
Supplemental Figure 5: Non-adherent cell death phenotype does not signal through apoptotic pathways. (A) Lysates from 1205LuTR LacZ and CADM1 cells were harvested in adherent and suspension conditions and used for analysis with apoptotic antibody array (RayBiotech #AAH-APO). (B) Western blots of indicated antibodies probing lysates of WM793TR CADM1, 1205LuTR CADM1, and A375TR CADM1 in adhesion and suspension conditions. (C) Representative stacked histograms of PI staining and quantified percentage of PI negative WM793TR LacZ and CADM1 cells in adhered and suspension conditions. Cells were treated doxycycline with or without 50uM olaparib for 24 hours prior to staining and analysis.

## Slide 6
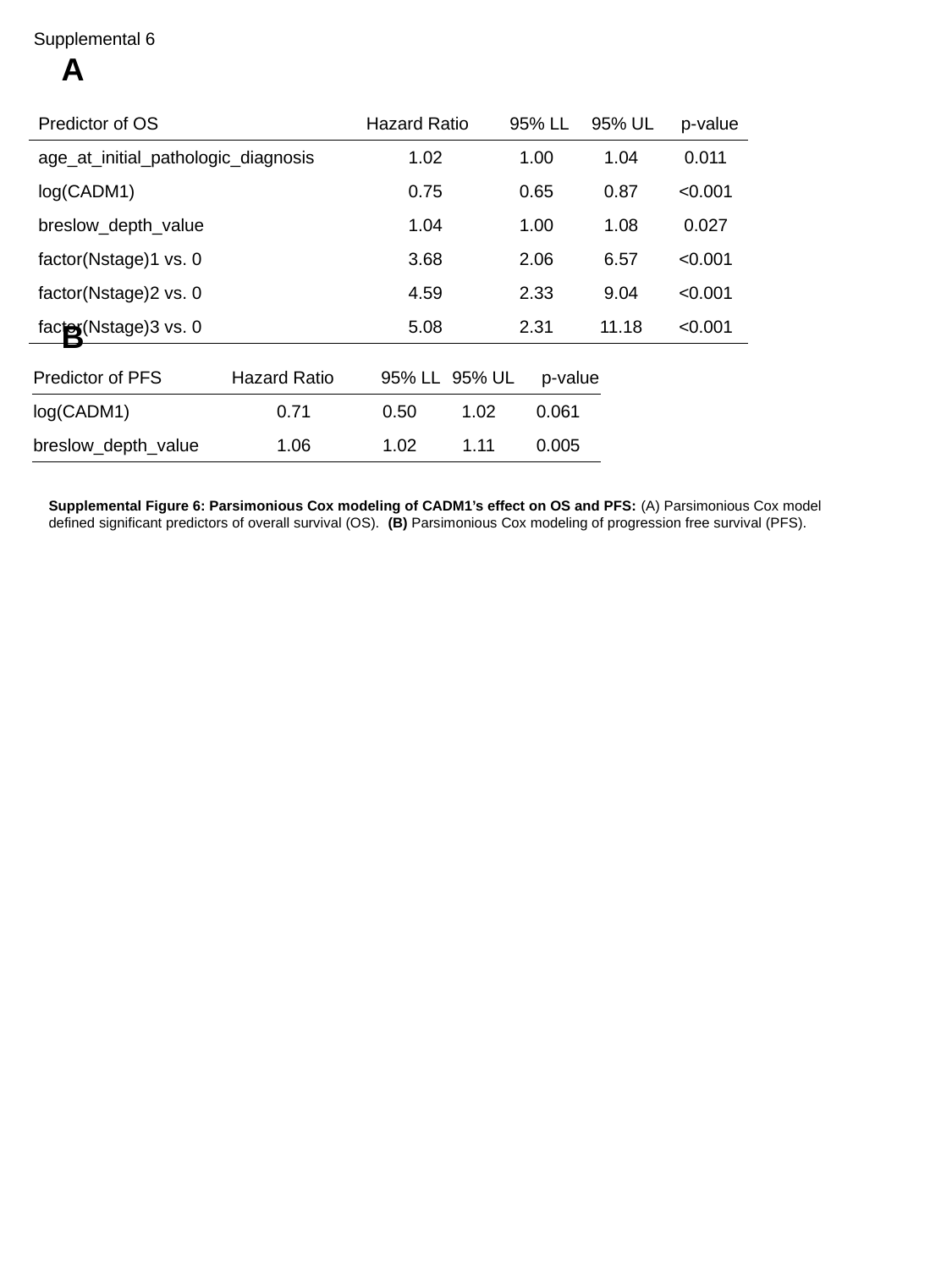

Supplemental 6
A
| Predictor of OS | Hazard Ratio | 95% LL | 95% UL | p-value |
| --- | --- | --- | --- | --- |
| age\_at\_initial\_pathologic\_diagnosis | 1.02 | 1.00 | 1.04 | 0.011 |
| log(CADM1) | 0.75 | 0.65 | 0.87 | <0.001 |
| breslow\_depth\_value | 1.04 | 1.00 | 1.08 | 0.027 |
| factor(Nstage)1 vs. 0 | 3.68 | 2.06 | 6.57 | <0.001 |
| factor(Nstage)2 vs. 0 | 4.59 | 2.33 | 9.04 | <0.001 |
| factor(Nstage)3 vs. 0 | 5.08 | 2.31 | 11.18 | <0.001 |
B
| Predictor of PFS | Hazard Ratio | 95% LL | 95% UL | p-value |
| --- | --- | --- | --- | --- |
| log(CADM1) | 0.71 | 0.50 | 1.02 | 0.061 |
| breslow\_depth\_value | 1.06 | 1.02 | 1.11 | 0.005 |
Supplemental Figure 6: Parsimonious Cox modeling of CADM1’s effect on OS and PFS: (A) Parsimonious Cox model defined significant predictors of overall survival (OS). (B) Parsimonious Cox modeling of progression free survival (PFS).
